# Medical Approach to Right Colon Diverticulitis with Perforation

**DOI:** 10.1155/2017/2563218

**Published:** 2017-10-02

**Authors:** James Espinosa, Rahul Sharma, Alan Lucerna, Doug Stranges

**Affiliations:** ^1^Department of Emergency Medicine, Rowan University-SOM/Kennedy University Hospital, Stratford, NJ, USA; ^2^Department of Surgery, Rowan University-SOM/Kennedy University Hospital, Stratford, NJ, USA

## Abstract

We report a case of a 71-year-old female who presented with right lower quadrant (RLQ) abdominal pain and was diagnosed on CT scan with right-sided diverticulitis with perforation. She was admitted under the surgical service after consultation and received intravenous fluids, intravenous antibiotics, and pain medications as needed. The patient was discharged 2 days after admission in stable condition with follow-up with gastroenterology. The differential diagnosis of right lower quadrant abdominal pain is vast. Right-sided diverticulitis often presents in a manner similar to appendicitis. In the absence of peritonitis, conservative treatment may be possible. It is predictable that as the population ages, the incidence of right-sided diverticular disease will increase and will result in more presentations of acute right-sided diverticulitis to the emergency department (ED). The aim of this case report is to increase awareness of the incidence, pathophysiology, presentation, work-up (laboratory studies and imaging), and management (medical and surgical) of right-sided diverticulitis among emergency physicians.

## 1. Introduction

Right-sided diverticulitis shares some elements in common with left-sided diverticulitis; however there are distinct features in reference to the incidence, pathophysiology, presentation, and management. In this case report, we will discuss those elements, partly in the context of comparison and contrast to left-sided diverticulitis and in the context of a 71-year-old female who presented with right lower quadrant (RLQ) abdominal pain and was diagnosed with right-sided diverticulitis with perforation.

## 2. Case Presentation

A 71-year-old presented to the emergency department (ED) with a complaint of right lower quadrant (RLQ) abdominal pain of one-day duration. She reported an episode of acute pain twelve hours prior to presentation, described as sharp in nature, which then became a constant aching pain. She denied nausea, vomiting, melena, hematochezia, or hematemesis. Sometimes in the past several years, the patient had been advised on a routine colonoscopy that she had diverticulosis. She never had an episode of diverticulitis. She also had a history of possible irritable bowel syndrome. She had no history of abdominal surgery. There was no family history of inflammatory bowel disease or gastrointestinal malignancies.

**Figure 1:**
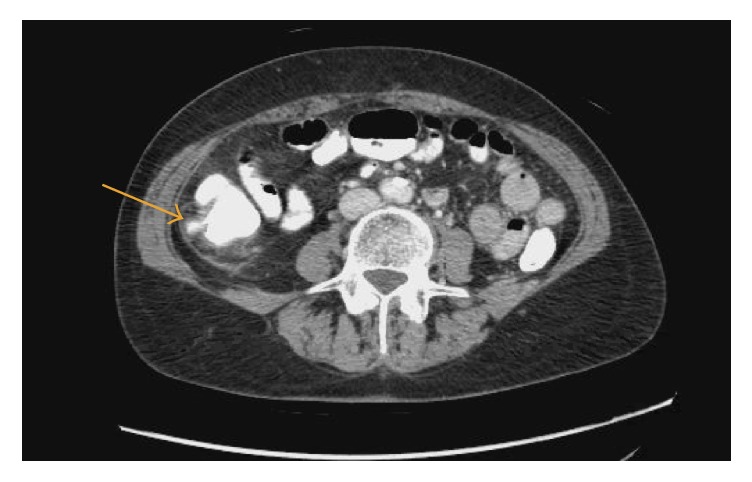
CT scan (axial view) showing an extension of contrast from the right posterior colon, cranial to the appendix.

**Figure 2:**
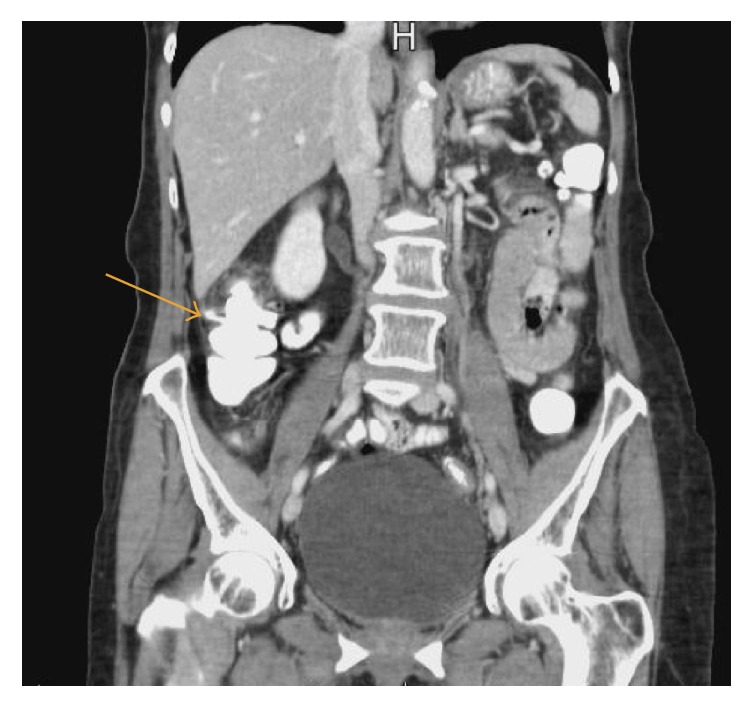
CT scan (coronal view) showing an extension of contrast from the right posterior colon, cranial to the appendix.

The patient's vital signs were as follows: heart rate of 99 beats per minute, respiratory rate of 18 breaths per minute, blood pressure of 143/76 mmHg, and a temperature of 97.9 degrees Fahrenheit, with a pain score of 6 out of 10. The physical exam showed diffuse right lower quadrant tenderness, with no rigidity, guarding. or rebound. Her white blood cell count was 12,900 per microliter with 77% neutrophils. Electrolytes and lipase were within normal limits. A computerized tomography (CT) scan of the abdomen and pelvis with oral and intravenous contrast showed an extension of contrast from the right posterior colon, cranial to the appendix. The appendix showed no evidence of inflammation, wall thickening, or dilatation. The axial and cranial views in tandem give a sense of the degree of extension of the contrast. The impression was of a perforated diverticulum or small contained rupture (Figures 1 and 2). Diverticula were noted throughout the colon.

The patient was seen in consultation in the ED by the surgical service. She was admitted to the hospital under the surgical service and received intravenous fluids, intravenous antibiotics (ertapenem, one gram intravenously daily), and pain medications as needed. A gastroenterology consultation was obtained.

The patient was discharged 2 days after admission in stable condition with follow-up with gastroenterology. Discharge medications included amoxicillin 500 mg three times per day for 7 days as per gastroenterology.

## 3. Discussion

The patient presented with right lower quadrant abdominal pain and tenderness that was demonstrated on CT scan to be due to right-sided diverticulitis with a perforated diverticulum.

### 3.1. Incidence of Right-Sided Diverticulitis

The incidence of right-sided diverticulosis and right-sided diverticulitis does not appear to be known [1]. It is noted in the literature to be rare [1–3]. As a general principle, diverticular disease increases with age. Diverticular disease is said to affect 50% of those aged 60 years and older and as many as 80% of the population aged 80 and older [4]. Many factors have been described as variables in the development, including dietary factors (such as decreased dietary fiber) as well as a lack of physical activity [5]. Genetics may play a part. This is evidenced by the fact that cecal diverticular disease has an incidence that is estimated to be 1 to 2% of surgical specimens in the North American population but approximately 43 to 50% of surgical specimens in studies of Asian populations [5]. The incidence of right-sided diverticulosis has been estimated by some to be approximately 8.5% of the population of western countries [6].

### 3.2. Pathophysiology

The pathophysiology of diverticulosis, right-sided or left-sided, is not yet completely understood. Such factors as colonic hypermobility, microflora content, and visceral hypersensitivity have been targets of research [5]. Other potential risk factors include aging, smoking, and alcohol intake [1]. Overall, most patients (80 to 90%) with diverticular disease are asymptomatic. Some cancers have been related to the development of diverticular disease, perhaps through the development of matrix metalloproteinase [5]. Multiple asymptomatic diverticula are a finding in older patients in association with carcinoma, while solitary diverticulum of the cecum may occur as a congenital lesion [6].

### 3.3. Presentation of Right-Sided Diverticulitis

Left-sided diverticulitis can present with left lower quadrant pain in association with such symptoms as nausea, vomiting, rectal bleeding, diarrhea, and fever.

Most patients with diverticula of the right side are asymptomatic [6]. Right-sided diverticulitis presents with right lower quadrant abdominal, nausea, and vomiting as well as other symptoms and thus can mimic appendicitis [1, 7–9]. Nirula and Greaney presented a series of 12 cases of right-sided diverticulitis over a 10-year period in a single hospital, in which the preoperative diagnosis of appendicitis was made in 11 cases. They recommended the consideration of right-sided diverticulitis when a normal appendix was found at the time of surgery for suspected appendicitis [10]. Violi et al. presented 20 cases of right-sided diverticulitis over a 22-year period. They conclude that right-sided diverticulitis should be considered in cases of atypical appendicitis presentations [11]. However, as well noted by Kahveci et al., the differential diagnosis of right lower quadrant pain “is vast” and includes not only acute appendicitis but also “ureteral colic, ectopic pregnancy, and a ruptured ovarian cyst.” [4]. Other forms of pathology may present with right lower quadrant pain and CT evidence of inflammation, including an inflammatory Crohn's mass, ileocecal tuberculosis, or a perforated foreign body reaction [1].

The initial presentation of diverticulitis can include bleeding, diverticulitis, peridiverticular abscess, and perforation [5]. An inflammatory colonic mass of uncertain etiology that is ultimately diagnosed as right-sided diverticulitis may have an initial working diagnosis of a carcinoma [8]. Complications of right-sided diverticulitis include “phlegmon, intra-abdominal abscess, fistulas involving adjacent organs, and distant septicemia” [7].

### 3.4. Laboratory Studies

Leukocytosis may be present in diverticulitis, but a normal white blood count does not rule out diverticulitis. Electrolyte abnormalities due to vomiting or diarrhea may be seen on a basic metabolic panel. Blood cultures should be obtained in patients who have complicated disease. Because a right-sided ectopic pregnancy may present with right lower quadrant abdominal pain, pregnancy should be ruled out in a woman of childbearing years with such a presentation.

### 3.5. Imaging

CT scanning appears to be the best overall imaging modality in the diagnosis of possible right-sided diverticulitis [5, 7–9]. “CT gives clinicians the most information about location and involvement of adjacent or distant organs or structures when compared to other imaging modalities” [7]. It was for this reason that a CT scan was used as the imaging modality of choice in this case. The two most common CT findings in diverticulitis are wall thickening and pericolic fat stranding, often in the context of an identifiably inflamed diverticulum [4]. MRI is an alternative imaging strategy that may be used in pregnancy or if necrotizing fasciitis is a diagnostic possibility [7]. Some studies have looked at the role of ultrasound in the evaluation of right lower quadrant pain [5]. According to Little and Culver, “the limitation of ultrasonography is the difficult in determining…whether or not colonic involvement is present or whether the abscess has penetrated the abdominal wall” [7].

### 3.6. Management

Karatepe et al. note that medical treatment may be sufficient in many patients with diverticulitis without peritonitis. Medical management consists of antibiotics targeted to treat common bacteria found in the colon, intravenous fluids, and pain medication [6, 7]. Our patient did not have signs of peritonitis and was able to be managed medically, while being carefully observed by the surgical service.

### 3.7. Surgical Treatment

In the absence of perforation and peritonitis, conservative treatment may be possible [9]. “Patients are typically managed medically first until complications…are apparent or are imminent” [7]. Surgical treatment may be a diverticulectomy [6]. Hildebrand et al. present their experience with 12 cases of right-sided diverticulitis over an 8-year period and concluded that surgery is only indicated in complicated right-sided diverticulitis and that “resection of the inflamed colon is safe and can be performed by laparoscopy” [3]. However, a right hemicolectomy may be needed if the specimen reveals colonic cancer [6].

## 4. Conclusions

The differential diagnosis of right lower quadrant abdominal pain is vast, but, in general, right-sided diverticulitis presents in a manner similar to appendicitis. In the absence of peritonitis, conservative treatment may be possible, as was the case in the patient presented. It is predictable that as the population ages, the incidence of right-sided diverticular disease will increase and will result in more presentations of acute right-sided diverticulitis to the emergency department.
